# Supplementary material for: Macroprudential policies and CO2 emissions: A comparative analysis of G7 and BRIC countries
Source: PLoS One. 2024 Jan 5;19(1):e0296363. doi: 10.1371/journal.pone.0296363 (PMC10769040; doi:10.1371/journal.pone.0296363)
Supplement: Attachment — Submitted filename: Response to Reviewers.docx [file pone.0296363.s003.docx]

PLOS ONE

Dear Editors and Reviewers:

Thank you for your letter and the reviewers’ comments concerning our manuscript (PONE-D-23-26694R1). Those comments are all valuable and very helpful for revising and improving our paper, as well as the important guiding significance to our research. We have studied comments carefully and have made corrections which we hope meet with approval. Revised portions are marked up using the “Track Changes” function in the paper. The main corrections in the paper and the responses to the editor’s and reviewer’s comments are as follows:

**Response to Reviewer 1 Comments:**

**Point 1:** Overall the revisions are good, but the language suggests further embellishment to improve the logic and readability of the whole text.

**Response 1:** We appreciate your valuable comments. The paper has been submitted for further editing, and we have also restructured our paragraphs for clarity and coherence.
